# Concerning Swimming

**Published:** 1881-11

**Authors:** 


### CONCERNING SWIMMING.

MANY good swimmers are of opinion that nobody need be drowned who does not lose his head when he falls into deep water. If he cannot swim he can float, it is maintained, or tread water. Against this theory may be set the experience of most men, that they can neither float nor tread water till they have learned how to swim. Both operations, both floating and treading, are natural and easy. But both require precisely that calmness and balance of mind and body which in the water are only possible to people who can trust their own powers. A swimmer leaves his body to the laws of nature, and presently his face and his toes are above the surface. But a man who cannot swim, as soon as he is out of his depth, thrusts up his arms —to clutch at he knows not what. Down goes his head, and by the time he has reached the surface again he has lost all nerve and forgotten all that he ever heard about the easiness of floating. We doubt whether Nelson, "who never felt fear," would have retained his calmness, if, being unable to swim, he had been thrown into deep water. That most men and women become idiotic with terror in these circumstances is demonstrated by their efforts to clutch and drown any one who comes to their assistance. The novelty of this new, implacable power, the water which is strangling them, drives all sense out of the heads of non-swimmers.

Trelawny and Shelley had been discussing these things, and Trelawny had expressed the belief, common to good swimmers, that all men can swim by gift of nature. Shelley never shrank from an experiment in morals, chemistry, or anything else, whence the frequent explosions that varied his career. He at once undressed and jumped into a deep place, sinking instantly to the bottom. There his slim body lay, still and glistening, and there the poet would have anticipated his fate if Trelawny had not dived in and dragged him to shore. If ever this experiment is to have a fair trial, it should be by immerging a very young child in water. Some people hold that he would swim as naturally as a puppy. But the maternal instinct, always unscientific, has hitherto stood in the way of this interesting experiment. In the present state of opinion on the subject it will be well for people to learn swimming of a competent teacher, either in a swimming bath, or in sea or river, before trying to rival Captain Webb by the mere light of nature. Every one who neglects to learn to swim should remember that he is endangering the lives of others, as well as his own. For it is seldom that a man is in danger of drowning that one of our countrymen is not found to peril his own life in the endeavor to rescue his neighbor.—*London News.*

